# Peste des Petits Ruminants Virus in Vulnerable Wild Small Ruminants, Iran, 2014–2016

**DOI:** 10.3201/eid2304.161218

**Published:** 2017-04

**Authors:** Mahmoud Marashi, Siamak Masoudi, Majid Kharazian Moghadam, Hossein Modirrousta, Mahyar Marashi, Masoumeh Parvizifar, Majid Dargi, Mahyar Saljooghian, Farbod Homan, Bernd Hoffmann, Claudia Schulz, Elke Starick, Martin Beer, Sasan Fereidouni

**Affiliations:** Department of Environment, Tehran, Iran (M. Marashi, S. Masoudi, M.K. Moghaddam, M. Marashi, M. Parvizifar, M. Dargi, M. Saljooghian, F. Homan);; Razi Research Institute, Karaj, Iran (H. Modirrousta);; Friedrich-Loeffler-Institute, Insel Riems, Germany (B. Hoffmann, E. Starick, M. Beer);; University of Veterinary Medicine Vienna, Vienna, Austria (S. Fereidouni);; University of Veterinary Medicine Hanover, Hanover, Germany (C. Schulz)

**Keywords:** peste des petits ruminants, PPR, peste des petits ruminants virus, wild goat, wild sheep, Iran, viruses

## Abstract

In 2014–2016, >1,000 wild goats and sheep in 4 northern and central provinces of Iran died from peste des petits ruminants virus (PPRV) infection. Partial nucleoprotein sequencing of PPRV from 3 animals showed a close relationship to lineage 4 strains from China. Control measures are needed to preserve vulnerable ruminant populations.

Peste des petits ruminants virus (PPRV; genus *Morbillivirus,* family *Paramyxoviridae*) causes a highly contagious disease with a high death rate in wild and domestic small ruminants. Four PPRV lineages (L1–L4) exist in Africa and Asia (*1*). The disease was initially recorded in Iran in 1995 (*2*) and subsequently spread throughout the country (*3*). PPRV-L4 infections are endemic in Iran and several neighboring countries (*4*,*5*).

Wild goats (*Capra aegagrus*) and sheep (*Ovis orientalis*), which have become extinct in several West Asia countries, are considered vulnerable species in Iran (*6*,*7*). Although PPRV-associated outbreaks among these ruminants have been suspected since 2000, the virus was not isolated or characterized at that time. In 2001, at least 1,500 wild goats and gazelles (*Gazella subgutturosa*) with clinical signs similar to those caused by PPRV infection died in Kavir National Park (Figure; Technical Appendix Table). An estimated 25%–40% of the wild goat population in the park was deemed lost as a result of the disease. In 2011, PPRV was the suspected cause of 550–700 deaths among wild sheep in Sarigol National Park (Figure); laboratory investigations using conventional reverse transcription PCR (RT-PCR) confirmed PPRV infection in several dead animals (Iran veterinary organization, pers. comm., 2011 Sep 26).

**Figure:**
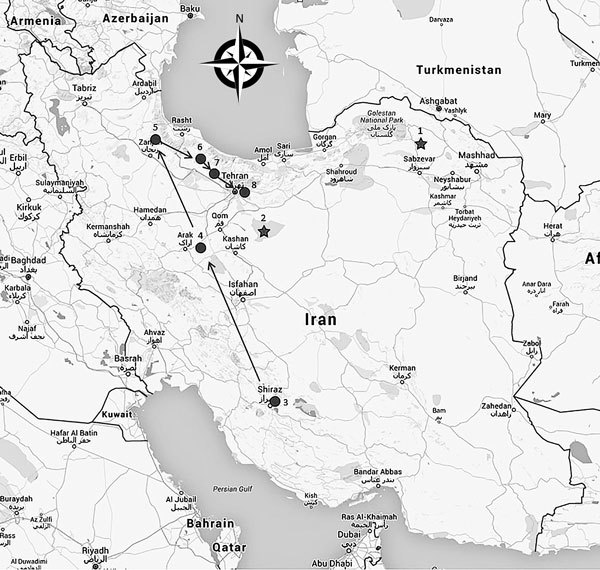
Geographic distribution of peste des petits ruminants virus outbreaks in Iran since 2000. Stars indicate outbreaks that occurred in 2000 and 2011; circles indicate outbreaks investigated during this study in 2014–2016. Arrows indicate chronologic order of the outbreaks: 1, Sarigol National Park; 2, Kavir National Park; 3, Bamou National Park; 4, Haftad-qolleh; 5, Kharmaneh Sar Tarom; 6, Alamout Protected Area; 7, Taleghan Protected Area; 8, Khojir National Park. Map generated using Google Maps (interactive map available at https://www.google.com/maps/d/viewer?mid=1GsluO7SZ2z_SBUawdPHsDF6s7ww). Details on the number of animals and dates of outbreaks are available in the Technical Appendix.

Beginning in September 2014, park rangers reported and field investigations substantiated mass deaths among wild goats in Bamou National Park (Figure). Clinical signs in affected animals were similar to those reported in wild small ruminants in 2011, and samples we tested from 5 dead goats were positive for PPRV by RT-PCR (online Technical Appendix). In April 2015, a new outbreak started in Haftad Qolleh Arak (Figure) and continued until mid-May, resulting in the death of 428 wild goats and 30 wild sheep. Three more outbreaks occurred in 2015: the first started in August in Kharmaneh-sar Tarom; the second in September in the Alamout Protected Area, 150 km from the previous outbreak in Kharmaneh-sar Tarom; and the third in November in the Taleghan Protected Area, 100 km away from the previous outbreak in Alamout Protected Area (Figure; online Technical Appendix Table).

The last reported outbreak started in April 2016 in Khojir, a national park close to a dam that serves as a water source for wild animals (Figure). In 2015, a total of 110 wild goats and sheep were counted in the park, and by May, 1, 2016, ≈85 were found dead (online Technical Appendix Table).

We detected PPRV genome in 6 oral swab samples and 7 blood and lymph node samples from dead ruminants by using conventional RT-PCR and in 3 oral swab samples by using real-time RT-PCR (quantification cycles 31–34) (online Technical Appendix). In addition, we performed partial nucleocapsid gene sequencing of 3 PPRV isolates from 2015; results showed 100% pairwise nt identity among the isolates (online Technical Appendix). The strains shared highest nt identity (99.4%) with PPRV-L4 strains that were circulating in domestic or wild small ruminants in northwestern and southeastern China during 2013–2015 (*8*) (online Technical Appendix Figure); they were more distantly related to PPRV-L4 strains previously reported from outbreaks in Iran and neighboring countries (*9*,*10*).

Field investigations and laboratory analyses indicated that PPRV was the cause of mass die-offs of wild goats and sheep during 2014–2016 in several national parks in Iran. A risk assessment of PPRV infection in several developing countries in Africa and the Middle East and on the Indian Peninsula indicated that 63% of small ruminant populations are at risk for infection (*4*). Legal and illegal movement of domestic small ruminants into wildlife territories over short and long distances, within and across borders, increases the possibility of transmission of various pathogens, including PPRV, to wild small ruminants, which may threaten vulnerable species. Transboundary circulation between China and Kazakhstan was recently shown for PPRV strains closely related to the PPRV Iran/2015 strains, suggesting that these closely related strains have been circulating in central and western Asia for a few years (*5*).

Clinical signs similar to those caused by PPRV infection were observed in domestic small ruminants in villages around the Kharmaneh-sar Tarom region before deaths were noted among wild goats in the area, and the samples collected from domestic animals tested positive for PPRV. It is unknown whether PPRV-infected wild small ruminants may contribute to PPRV spread by spillback to domestic small ruminants.

Comprehensive field studies of PPRV infection in domestic and wild small ruminants are necessary to evaluate the occurrence and origin of PPRV infections and of different PPRV strains in domestic and wild small ruminants in Iran. Emerging PPRVs can potentially spread to all susceptible small ruminant populations in the region and cause extinction of local subpopulations. Furthermore, control measures, such as vaccination against PPRV and movement control of domestic small ruminants around protected areas, would facilitate the preservation of vulnerable wild small ruminant populations and reduce the economic effect of PPRV infection on small ruminant production in affected regions.

Technical Appendix Materials and methods used to characterize the peste des petits ruminants viruses (PPRV), and location and number of dead wild goats and wild sheep and phylogenetic tree of PPRV strains, Iran, 2014–2016.
