# Supplementary material for: Peste des Petits Ruminants Virus in Vulnerable Wild Small Ruminants, Iran, 2014–2016
Source: Emerg Infect Dis. 2017 Apr;23(4):704–6. doi: 10.3201/eid2304.161218 (PMC5367395; doi:10.3201/eid2304.161218)
Supplement: Technical Appendix — Materials and methods used to characterize the peste des petits ruminants viruses (PPRV), and location and number of dead wild goats and wild sheep and phylogenetic tree of PPRV strains, Iran, 2014–2016. [file 16-1218-Techapp-s1.pdf]

Article DOI: http://dx.doi.org/10.3201/eid2304.161218

Peste des Petits Ruminants Virus in Vulnerable Wild Small Ruminants, Iran, 2014–2016

Technical Appendix

Materials and Methods

Within the framework of outbreak investigation, a standard operating protocol of field epidemiologic data collection, post-mortem examination of carcasses, sample collection and submission, and laboratory analyses were carried out in this study.

RNA was extracted from oral swabs, tissue (lymph node and spleen) samples and whole blood samples obtained from more than 22 dead wild goats/sheep (found during last six outbreaks between September 2014 and April 2016, Table 1) using RNeasy® Mini Kit (Qiagen) and QIAmp® Viral RNA Mini Kit (Qiagen).

The samples were analyzed for PPRV-RNA using conventional RT-PCR (*1*) and real-time RT-PCR (SensiFAST Probe No-ROX Kit, Bioline) based on a published PCR-assay (*2*) that detects partial nucleocapsid protein (N) of PPRV. PPRV genome was detected in 6 oral swabs and 7 blood and lymph node samples using conventional RT-PCR and later in 3 oral swabs using real-time RT-PCR (quantification cycle (C_q) values: 31 to 34).

Partial PPRV nucleocapsid (N) sequences of 3 real-time RT-PCR-positive oral swabs of a length between 948 and 1097 bp were obtained with published (*3*–*5*) and unpublished (available on request) forward and reverse primers and used for sequencing. A pairwise identity of 100% was found between the three PPRV-RNA positive samples. Since only part of the PPRV genomes was sequenced, we cannot exclude that the three samples may contained three different, but very closely related PPRV strains. Three representative sequences was compared to a selection of PPRV sequences available in the GenBank (http://www.ncbi.nlm.nih.gov/genbank)

and used for phylogenetic analysis (Figure 1). The representative sequence is available with GenBank accession number KY550670.

References

1. World Organisation for Animal Health (OIE). Peste des petits ruminants. In: Manual of standards for diagnostic tests and vaccines for terrestrial animals. Paris: 2013 [cited 2016 Jul 15]. http://www.oie.int/fileadmin/Home/eng/Health_standards/tahm/2.07.10_PPR.pdf

2. Bao J, Li L, Wang Z, Barrett T, Suo L, Zhao W, et al. Development of one-step real-time RT-PCR assay for detection and quantitation of peste des petits ruminants virus. J Virol Methods. 2008;148:232–6. PubMedhttp://dx.doi.org/10.1016/j.jviromet.2007.12.003

3. Polci A, Cosseddu GM, Ancora M, Pinoni C, El Harrak M, Sebhatu TT, et al. Development and Preliminary Evaluation of a New Real-Time RT-PCR Assay For Detection of Peste des petits Ruminants Virus Genome. Transbound Emerg Dis. 2015;62:332–8. PubMedhttp://dx.doi.org/10.1111/tbed.12117

4. Batten CA, Banyard AC, King DP, Henstock MR, Edwards L, Sanders A, et al. A real time RT-PCR assay for the specific detection of Peste des petits ruminants virus. J Virol Methods. 2011;171:401–4. PubMedhttp://dx.doi.org/10.1016/j.jviromet.2010.11.022

5. Kwiatek O, Keita D, Gil P, Fernández-Pinero J, Jimenez Clavero MA, Albina E, et al. Quantitative one-step real-time RT-PCR for the fast detection of the four genotypes of PPRV. J Virol Methods. 2010;165:168–77. PubMedhttp://dx.doi.org/10.1016/j.jviromet.2010.01.014

Technical Appendix Table. Location and number of dead wild goats and wild sheep associated with PPRV outbreaks reported in domestic small ruminants in Iran since 2000*

Year	Month	Outbreak region	Total no. dead	No. wild goats	No. wild sheep	No. gazelle
2001	May–Jun	Kavir National Park[1]	1,500–2,500	×		×
2011	Aug/Sep	Sarigol National Park[2]	550–700	–	550–700	–
2014	Sep–Nov	Bamou National Park[3]	400	×	×	–
2015	Apr/May	Haftad-qolleh[4]	458	428	30	–
2015	Aug/Sep	Kharmaneh sar tarom[5]	126	126	–	–
2015	Sep–Nov	Alamout Protected Area[6]	30	30	–	–
2015	Nov–Dec	Taleghan Protected Area[7]	204	204	–	–
2016	Mar–Apr	Khojir National Park[8]	85	85	–	–

*x, no. of dead species not exactly defined; –, dead species not reported; [1–8], numbers correspond to outbreak areas on the map in main text.

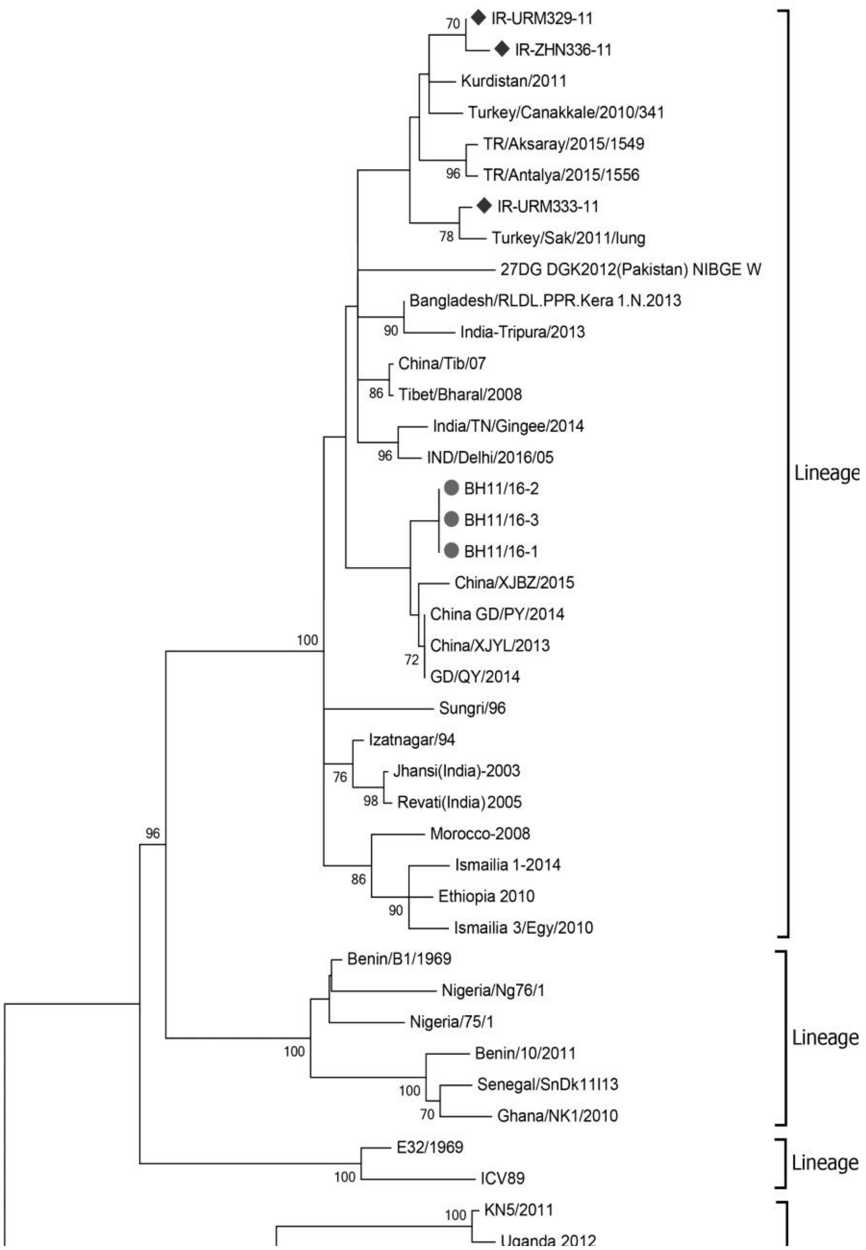

Technical Appendix Figure. Phylogenetic tree of Peste des Petits Ruminants viruses (PPRV) lineages 1 to 4 based on the N gene, including the "PPRV Iran/2015" strains (red circle marked) obtained from three wild goats in this study (laboratory submission numbers BH11/16–1 to –3) and isolates previously reported from PPRV outbreaks in Iran (blue marked). The closest relationship of PPRV Iran/2015 was found with PPRV strains from the provinces Xinjiang (China/XJBZ/2015 and China/XJYL/2013) and Guangdong (China GD/PY/2014 and GD/QY/2014) in northwestern and southeastern China, respectively. The tree was constructed using maximum-likelihood approach (MEGA 6.0 software) based on the GTR+I+G model. Numbers indicate the bootstrap values of 1000 replicates (only values >75% are shown) and the scale bar nucleotide substitutions per site.
